# Biases in the SMART-DNA library preparation method associated with genomic poly dA/dT sequences

**DOI:** 10.1371/journal.pone.0172769

**Published:** 2017-02-24

**Authors:** Oriya Vardi, Inbal Shamir, Elisheva Javasky, Alon Goren, Itamar Simon

**Affiliations:** 1 Department of Microbiology and Molecular Genetics, IMRIC, The Hebrew University Hadassah Medical School, Jerusalem, Israel; 2 Department of Medicine, University of California San Diego, La Jolla, CA, United States of America; German Cancer Research Center (DKFZ), GERMANY

## Abstract

Avoiding biases in next generation sequencing (NGS) library preparation is crucial for obtaining reliable sequencing data. Recently, a new library preparation method has been introduced which has eliminated the need for the ligation step. This method, termed SMART (switching mechanism at the 5′ end of the RNA transcript), is based on template switching reverse transcription. To date, there has been no systematic analysis of the additional biases introduced by this method. We analysed the genomic distribution of sequenced reads prepared from genomic DNA using the SMART methodology and found a strong bias toward long (≥12bp) poly dA/dT containing genomic loci. This bias is unique to the SMART-based library preparation and does not appear when libraries are prepared with conventional ligation based methods. Although this bias is obvious only when performing paired end sequencing, it affects single end sequenced samples as well. Our analysis demonstrates that sequenced reads originating from SMART-DNA libraries are heavily skewed toward genomic poly dA/dT tracts. This bias needs to be considered when deciding to use SMART based technology for library preparation.

## Introduction

Next generation sequencing (NGS) technologies have become a major research tool in all fields of biology [1]. Massive sequencing is important for many fields including comparative genomics [2–4], human population genetics, personalized medicine [5, 6] and microbiome research [7]. In addition, the relatively low cost of NGS makes it the method of choice for many genomic measurements including genome-wide levels of RNA (RNA-seq), localization of DNA-associated proteins (ChIP-seq), and DNA methylation. Moreover, new applications for NGS are constantly being developed, expanding the ability to measure all kinds of genomic and transcriptomic features [8]. All these applications include the addition of known sequences to both ends of the nucleic acid material (DNA or RNA) that are then used both for amplification and for sequencing.

The underlying assumption of all these methods is that no major biases are introduced during library preparation and that the composition of the library reflects the composition of the initial representations of the DNA or RNA molecules. However, various biases are introduced during library preparation. This topic was recently reviewed by [9], which concluded that the major steps in DNA library preparation that introduce biases are: size selection [10], PCR amplification [11–14], and chromatin fragmentation in the case of ChIP-seq experiments [15]. Notably, during DNA library preparation, there is no evidence of biases being introduced during adapter ligation.

**Fig 1:**
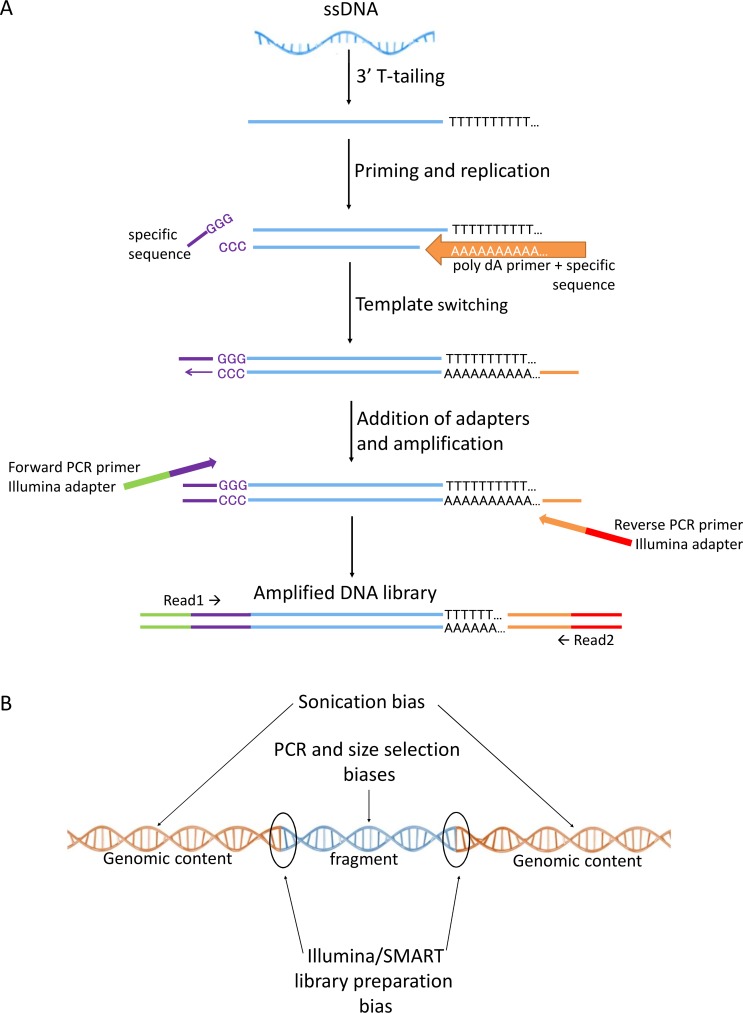
Template switching protocol and type of biases in NGS. (A) Schematic representation of the SMART library preparation method comprised of: poly dT tailing, priming and second strand synthesis, template switching by MMLV-RT, and addition of adapters and PCR amplification. (B) Schematic representation of the potential biases resulting from the different steps of the library preparation method. The effect of the bias may occur in the sequenced fragment (due to PCR and size selection), in the genomic content of the fragment (due to sonication) or in the interface between the fragment and the genomic content (due to the different methods of adapter addition).

Although the ligation step is not known to introduce biases, it is not efficient and thus a typical NGS protocol requires high quantities of starting material. Recently, this problem was overcome by replacing the inefficient ligation step with a template-switching based mechanism [16]. Indeed, this methodology, frequently termed SMART (for Switching Mechanism At the 5′ end of the RNA Transcript) allows library preparation from very small amounts of RNA and was shown to give reliable results for single cell library preparation [17–23]. The SMART methodology was initially developed for RNA library preparation. It takes advantage of the available poly dA tail of mRNAs by annealing an adapter sequence with a poly dT tail. Moloney murine leukemia virus reverse transcriptase uses this primer to copy the RNA strand. When the reverse transcriptase reaches the 5’ end of the RNA template, the enzyme’s terminal transferase activity adds three non-templated cytosine residues to the cDNA. The second adapter which has a poly dG tail, base-pairs with these additional non-template nucleotides and creates an extended template, enabling the reverse transcriptase to continue replicating to the end of the oligonucleotide. Sequencing libraries are then generated by PCR-mediated addition of Illumina adapters using primers compatible with regions on the poly dT and the poly dG oligonucleotides (Fig 1A) [16, 24, 25]. More recently, this methodology has been adopted for DNA library preparation by adding a poly dT or poly dA sequence to the 3’ end of the DNA template using the terminal deoxytransferase (TdT) [25].

The replacement of the ligation step by template switching may introduce additional biases at the sequences adjacent to both ends of the reads, while it should not affect other types of biases (Fig 1B). However, this source of bias has not been studied extensively. To the best of our knowledge, only one aspect of the biases of the SMART library preparation procedure has been investigated; Tang et al., pointed out that the template switching mechanism is susceptible to strand invasion, which may cause an enrichment of RNA sequences with inner GGG. This bias is especially pronounced when the template switching procedure is used to add indexes to the samples [26]. Here we perform a systematic analysis of the genomic content of reads obtained from DNA template switching based libraries. We found that a large portion of the reads (>16%) ended adjacent to poly dA or poly dT tracts, a phenomenon which can be easily explained by the SMART protocol.

## Results

### Information content analysis

In order to investigate the specific biases introduced into the sequencing results by the template switching protocol, we compared sequencing results between template switching and ligation based libraries. To this end, we harvested human genomic DNA (HCT116 cells), sonicated it, and sequenced it using either the DNA SMART ChIP-Seq Kit (Clontech), (which utilizes the template switching methodology for library preparation), or a conventional ligation mediated library preparation method (see methods). Both libraries were sequenced using the Illumina paired end sequencing protocol and aligned to the human genome (2X50; 1-6x coverage; S1 Table, all PCR duplicates were removed from the data prior to further analyses). The sequencing of the ligation mediated libraries initiates from primers that recognize sequences at the end of the adapters. On the other hand, the SMART based library protocol uses a special second read sequencing primer that contains poly dA at its 3’ end in order to avoid sequencing the poly dT added to the end of each fragment. While this sequencing strategy helps in eliminating non-genomic poly dT, it also eliminates genomic poly dT, which appears outside of the sequenced fragment as part of the genomic content.

**Fig 2:**
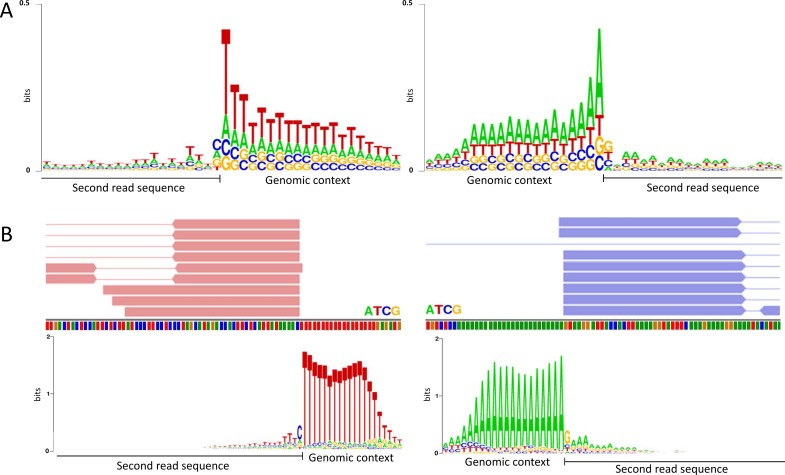
Base constitution surrounding the 3’ end of the sequenced fragments in SMART based libraries. (A) Sequence logo representation of the information content of the region surrounding the end of the second read for the forward (left) and reverse (right) strands in a HCT116 sample prepared with the SMART based library protocol (S1 Table). Similar results were obtained with all other SMART based samples. Each sequence logo is based on 1,000 randomly chosen reads. (B) IGV representation of two typical genomic regions (taken from the same SMART based library as in A), in which multiple reads ended at the same position. The red and blue rectangles represent the locations of the second reads in each pair for the forward (left) and the reverse (right) strands respectively. The small bars below the reads represent individual bases which are color coded. Note that immediately after the reads there are tracts of poly dT and poly dA for the forward and reverse strands, respectively. The sequence logos below the IGV tracts represent the information content as in A, for 1,000 randomly chosen genomic regions out of ~300,000 in which at least 5 reads were mapped.

We calculated the information content of both ends (the beginning of the first read and the end of the second read in each pair) of all the libraries. We found no nucleotide preferences at either end of the ligation-based protocol. In contrast, in the SMART based protocol, although there was no nucleotide preference at the beginning of the fragment, we found a strong nucleotide bias at the end of the fragments. The bias is remarkably strand-specific; in the forward strand, it is toward T, whereas in the reverse strand it is toward A (Fig 2A, S1 Fig). The difference between the strands is not surprising, given that SMART based library preparation retains strand information. Thus, for example, poly dT at the end of the forward strand will be mapped to poly dT sequences, whereas the same sequence on the reverse strand will be mapped to their reverse complement sequences, which are poly dA.

By inspecting the genomic alignments of all mapped fragments, we found that there are many genomic regions in which several read ends were mapped at exactly the same position (Fig 2B). These regions were almost always located next to long (n≥12) poly dT or poly dA (depending on the strand) tracts. Indeed, the information content at enriched (containing ≥5 second reads with the exact same position) genomic regions was heavily biased toward T and A for the forward and reverse strands respectively (Fig 2B).

### Bias toward poly dA/dT genomic tracts

These results suggest that the SMART- DNA library preparation methodology has a bias toward poly dA/dT tracts. This is not surprising since the SMART protocol uses poly dT tracts, added by the TdT enzyme, for adapter annealing. Thus, it may prefer genomic regions that contain such tracts and are ready for library preparation even without the activity of the TdT enzyme. It should be noted that this model explains the different nucleotide preferences between the strands. While in both strands poly dT will be recognized by the poly dA containing adapter, in the reverse strand, poly dT will be mapped to poly dA since the reported sequence is always from the forward strand.

**Fig 3:**
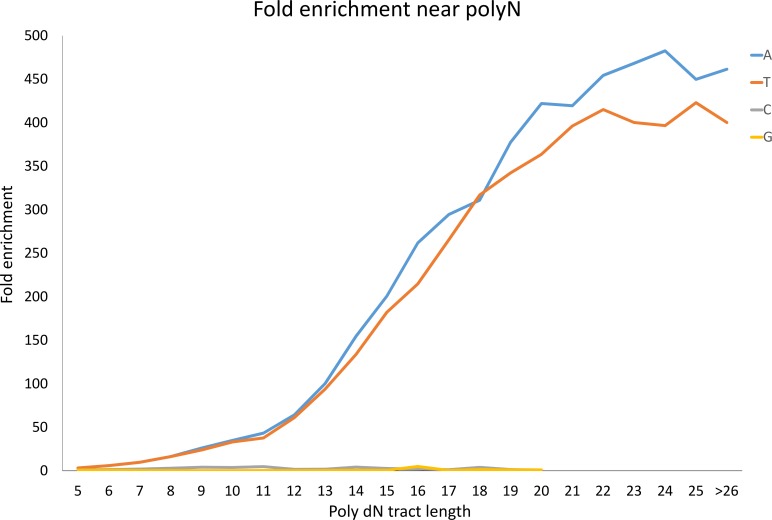
Poly dN tract length analysis in a SMART based library. Graph representing the ratio between the number of reads in the forward strand versus the number of reads in the reverse strand that were adjacent to various lengths of poly N tracts. The different colors represent the four nucleotides. For T, C and G we present the forward/reverse ratio whereas for A the opposite ratio (reverse/forward) is presented.

Poly dA and poly dT tracts occur at a similar frequency in the genome and are much more abundant than poly dC and poly dG tracts (for example, there are approximately 100 times more poly dA and poly dT tracts of at least 10 nucleotides than poly dC and poly dG; S2 Fig). This is probably due to the genomic integration of RNA transposable elements, which contain poly A tracts [27]. We checked the over representation of reads (in the SMART based libraries) which either overlapped or ended exactly adjacent (distance = 1bp) to poly dT tracts and compared it to the frequency of reads ending at poly dA tracts (in the forward strand). We found a bias toward poly dT tracts when the size of the tract was as small as n = 5 (3.4 fold), it increased gradually until n = 11, and became much more pronounced starting at n = 12 (61 fold). This bias increased until n = 21 (~400 fold) where it reached a plateau. Similar over representation was found in the reverse strand toward poly dA (Fig 3). Thus, we decided to perform all of the following analyses on poly dN tracts of at least 12 nucleotides.

**Fig 4:**
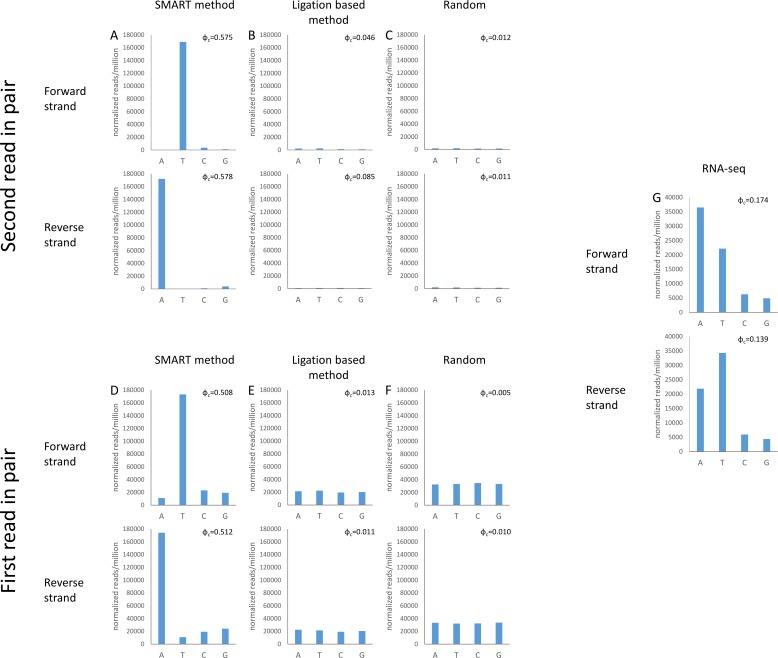
Bias toward Poly dT tracts in SMART-DNA libraries. The number of reads (per million reads) mapped adjacent to poly dN tracts (≥12) for the forward and reverse strands is reported. These numbers are further normalized for the genomic frequencies of such tracts (see methods). Data is shown for the second read in each pair (A-B), for the first read (D,E,G) and for random locations (shift of 1000 bps, see methods) (C,F). The analysis was done for SMART-DNA based library preparation (A,D), for ligation based method (B,E) and for SMART-RNA library preparation (G). The distance between the read to the poly dN tract was set to be either 1 nucleotide (A-C) or 250 nucleotides (D-G). The data presented is for HCT116 genomic DNA sequenced by us (A, D), obtained from the Aladgem lab (B,E) and for RNA seq libraries (G) downloaded from [17]. Note that the SMART-RNA library was prepared by annealing a primer to poly dA tracts rather than to poly dT tracts as with other SMART based libraries. We tested the statistical significance of the deviation from a distribution that is based on the frequency of occurrences in the genome, using the Chi squared goodness of fit test. The effect sizes (ϕ) (see methods) are shown in each graph. The data presented is for a single library from each type. Similar results were obtained for additional libraries (S3 Fig).

We looked at the number of occurrences of a read next to poly dN and found considerable enrichment for poly dT or poly dA (depending on the strand) versus poly dC and poly dG. Actually, approximately 16% of the reads ended exactly adjacent to poly dT or poly dA tracts (n≥12) for the forward and reverse strands respectively, while almost no reads were located adjacent to poly dC and dG (n≥12; 2.1x10^-5^%, 1.5x10^-5^% and 1.4x10^-5^%, 2.8x10^-5^% for the forward and reverse strands respectively). We further normalized the data for the genomic abundance of each poly dN tract (n≥12) and found that even after normalization, the bias toward poly dT on the forward strand and poly dA on the reverse strand was still quite substantial. This bias did not occur in ligation-based libraries or in randomized data (Fig 4).

The described bias toward poly dT tracts was obvious only when inspecting the second read in each pair (Fig 2). While more than 15% of the second reads were located next to poly dA/dT tracts (Fig 4A), only approximately 0.001% of the first reads were adjacent to poly dA/dT tracts. Nevertheless, this bias affects various SMART-DNA based experiments regardless of the sequencing protocol (both SE and PE sequencing). Examining the abundance of first reads that were located in the vicinity (up to 250 bases) of poly dN tracts revealed a strong bias toward poly dA/dT (Fig 4D). This bias is indeed unique to the SMART based library preparation protocol and does not exist in ligation-mediated protocol or in randomized data (Fig 4E and 4F).

In order to confirm the generality of the bias toward poly dA/dT tracts in SMART based libraries, we have repeated the analyses on two additional SMART based libraries, four additional ligation based libraries, and four additional RNA-seq datasets, and received almost identical results (S3 Fig).

## Discussion

Ligation free protocols for library preparation have several advantages. First, they use very small amounts of starting material, thus allowing the study of the transcriptome of single cells [17–23, 25]. Second, they enable strand specific library preparation for both RNA and DNA samples. Finally, the protocols are much simpler than the ligation mediated protocols, reducing library preparation time to a few hours. However, our analyses demonstrate that these protocols introduce a new type of bias. While the ligation mediated DNA library preparation protocols suffer mainly from biases related to sonication and PCR amplification, the template switching based protocols introduce additional biases stemming from the preferential amplification of fragments containing poly dT.

This type of bias is most probably a consequence of the need for the presence of poly dT at the 3’ end of each fragment for the annealing of the poly dA primer (S4A Fig). Ideally, poly dT is added to each fragment by the terminal deoxytransferase (TdT) enzyme prior to the annealing step and thus all fragments should have an equal chance of being annealed. However, if the TdT enzyme does not add the poly dT to all fragments there will be a bias toward sequences that already contain poly dT as part of their genomic sequence. Notably, even if the genomic poly dT is located within the fragment and not at its 3’ end, it can be annealed to the poly dA primer. Indeed, fragments ending at poly dA/dT genomic locations were significantly shorter than fragments ending in all other genomic locations (average lengths were 186.5 versus 223.3 respectively; P value < 2.2e-16, t test).

We have explained the bias toward poly dA/dT tracts as a consequence of biased library preparation (S4A Fig). Theoretically, the bias could occur during sequencing, due to the use of a special sequencing primer which may favor internal sequences that contain poly dAs, or during the mapping process, which may shift the location of the reads closer to genomic poly dA/dT tracts (S4B and S4C Fig). However, we do not think that either of these alternative explanations would account for the observed bias toward poly dA/dT tracts. First, by running a simulation using random genomic fragments with a similar length distribution to that of the actual sequenced fragments, we found that less than 4.5% of the simulated fragments contained poly dA/dT tracts, which is significantly less than the observed 16%. Second, we used the end-to-end option for mapping, which uses the entire sequence of the read for mapping. This mapping approach may cause failures when mapping reads that contain non-genomic poly dAs at their 3’ end. In order to explore this possibility we remapped the reads after trimming 10 bases from the 3’ end of the second read. The overall alignment rates were essentially the same (77.44% before trimming versus 78.2% after), demonstrating that non-genomic sequences at the 3’ of the reads have an insignificant effect on mapping. Finally, the mapping procedure only introduced gaps in a very small percentage of reads (1.46%) verifying that mis-mapping cannot explain the extensive bias toward poly dA/dT tracts.

The annealing of the 5’ adapter is mediated by three cytosines added to the 5’ end of the fragment by the Moloney murine leukemia virus reverse transcriptase (Fig 1). We did not find any nucleotide enrichment in the 5’ end of the first read (S1 Fig), indicating that these three Cs did not cause any apparent bias. Interestingly, Tang et al., did find 5’ biases when they compared libraries prepared with various 5’ adapters for barcoding. They reasoned that these biases were a consequence of strand invasion [26] that occurred due to the use of barcode containing adapters. In our datasets, the 5’ adapter used did not contain a barcode, and therefore it was probably too short to cause strand invasion.

The most common application of the SMART library preparation method is for RNA-seq. In such protocols there is no use of the TdT enzyme and the annealing is usually based on the natural poly dA tracts at the end of mRNA molecules[16]. Thus, the only potential source of bias could be caused by internal poly dA that are annealed by the primer. Indeed, analysis of RNA-seq data produced by the SMART-RNA protocol [17] revealed a small but significant bias toward poly dA in the forward strand (Fig 4H).

The described strong bias toward genomic poly dT tracts in SMART-DNA libraries makes it difficult to rely on their results. The main use of the SMART-DNA library preparation protocol is for ChIP-seq experiments. When analysing ChIP-seq data, a peak-calling algorithm is used to identify protein-binding sites. Most of these algorithms take advantage of input DNA to correct for biases in specific genomic regions (such as repetitive sequences). Nevertheless, these algorithms rely on many assumptions and may fail to correctly deal with the poly dA/dT bias. It is for this reason that we strongly discourage using SMART based library preparation for DNA samples, unless other options are not viable (such as in cases of very little amounts of starting material). In such cases, where the SMART based protocol is the best choice, extensive data filtering is required.

Data randomization revealed that only 1% of the reads mapped to clusters of at least 5 reads ending at the exact same genomic position. However, in the SMART-DNA libraries, we found that ~20% of the reads were mapped to such clusters. Filtering all reads mapped adjacent to poly dA/dT tracts (≥12) reduced this number to ~7%, which is still much higher than the expected 1%. It appeared that many of the reads clustered next to poly dA/dT tracts that contained a mismatch. Indeed, filtering out all reads adjacent to poly dA/dT tracts (≥12) with up to two mismatches reduced the number of reads that were clustered to 2% of the reads.

Based on this analysis, we suggest omitting all reads adjacent to poly dA/dT tracts (≥12) with up to two mismatches. Although this filtering strategy removes most of the biases, it does so at the expense of reducing the total number of mappable reads (26% of the reads were filtered out) and introduces a different bias, against genomic regions adjacent to poly dA/dT tracts. A better solution is to modify the library preparation protocol in a way that would avoid such biases. We suggest two possible modifications to the protocol that should solve this problem. First, instead of using the TdT enzyme to add dTs, the exact same enzyme can be used to add dGs [28]. This will reduce the bias significantly since poly dGs are much less abundant in the human genome (S2 Fig). A similar solution would be to avoid the use of the TdT enzyme altogether, and to base primer annealing on random primers as has been done in some SMART-RNA protocols [29]. Undoubtedly, these protocol modifications may introduce new biases that would have to be investigated further.

## Conclusions

We have described a severe bias toward genomic poly dA/dT tracts that exists in sequencing results of libraries prepared by the SMART-DNA methodology. This bias is apparent both in single end and in paired end DNA-seq libraries, and to a lesser extent in RNA-seq libraries. This bias could affect any counting based protocol such as ChIP-seq, CNV determination, Hi-seq, ATAC-seq, etc. RNA-seq data is also affected (although to a lesser extent) by SMART based technologies, and thus, wherever possible, SMART based libraries should be avoided for RNA-seq as well. The effects of this bias can be diminished by filtering the data, but this results in a reduction of the effective library size by 26%. Future improvements in the SMART protocol are needed to overcome these biases.

## Material and methods

### Cell growth

HCT116 cells were grown in McCoy’s medium (biological industries #01-075-1); supplemented with 50 g/ml Penicillin-Streptomycin (biological industries # 03-031-1), 2mM L-glutamine (biological industries # 03-020-1), 2mM pyruvate (biological industries # 03-042-1) and 10% Fetal Bovine Serum (FBS) (biological industries # 04-127-1). HeLa-S3 cells were grown in a spinner flask in DMEM medium with 0.1% pluronic F-68 (Sigma # 9003-11-6), 50 g/ml Penicillin-Streptomycin, 2mM L-glutamine, 2mM pyruvate and 10% FBS. All cell lines were grown in a humidified 370C incubator with 5% CO2.

### Library preparation and sequencing

Overall, we analysed 8 DNA libraries (7 of which we prepared and sequenced ourselves; S1 Table). DNA harvested from HCT116 cells was either sonicated (Bioruptor) or fragmented to 400–800 bps using a sucrose gradient [30]. The sheared DNA was used for library preparation using the DNA SMART ChIP-Seq Kit (Clontech, #634865). Each library was prepared from independently made DNA.

This data was compared to libraries prepared with an Illumina based protocol. To this end, we used either an HCT116 library obtained from the Mirit Aladjem laboratory, or HeLa-S3 DNA libraries which were sonicated with Covaris and independently prepared as described [31, 32]. Briefly, the DNA was end repaired using Klenow (NEB #M0212M) and ligated to Illumina paired-end adapters (TruSeq). The ligated libraries were size selected (in order to remove free adapters), PCR amplified, and purified with SPRI beads (Agencourt AmPure beads, A63881, Beckman Coulter).

DNA samples were sequenced using paired end Illumina NextSeq or HiSeq technologies. For the SMART based libraries, the second read was sequenced using a custom sequencing primer provided by the library preparation kit (Clontech). A summary of all samples used, along with descriptions of the basic sequencing features, are shown in S1 Table.

Four libraries of RNA-seq data prepared independently by the SMART protocol [17] were also analysed (GSM967491, GSM967511, GSM967559 and GSM967577).

### Bioinformatics analyses

In a regular library preparation the sequencing primers ensure that the resulting sequence is almost identical to the genomic fragment sequenced (apart from sequencing errors). On the other hand, in SMART based libraries, three nucleotides are added at the 5’ of each fragment and poly dTs are added at its 3’ (Fig 1). In order to account for these non-genomic nucleotides, the manufacture suggests omitting the first nucleotides from every first read, and using a special sequencing primer that contains poly dT so the actual sequence will start beyond the artificially added poly dAs. Thus, for SMART based libraries we trimmed the first four nucleotides before mapping, while for Illumina based libraries we used the entire sequence. All reads were mapped to the hg19 reference genome using Bowtie2 [33] using default parameters with the maximum fragment length for valid paired-end alignments set at 1000. Only reads that were mapped to a single genomic location were used for further analyses. PCR duplicates were removed using the Picard tools MarkDuplicates software (http://broadinstitute.github.io/picard). Genomic locations with very large numbers of reads (>100) which likely represent repetitive sequences, were also filtered from the data.

Poly dN genomic locations were identified using the fuzznuc program (from EMBOSS [34]). BEDtools [35] and SAMtools [36] commands were used to determine overlaps between reads and genomic poly dN tracts (within a distance of 1 base using the following commands—bedtools window -r 1 -l 0 -u and bedtools window -r 0 -l 1 –u, for the forward and reverse strands respectively). The Information content of the 20 bp surrounding each end of every read was calculated using the WebLogo tool [37], due to limitations of this tool we randomly chose 1,000 reads for drawing the webLogo. We repeated this procedure five times for each library and received essentially the same results. The number of reads (per million sequencing reads) overlapping or located immediately adjacent (distance = 1bp) to a poly dN tract were counted for each type of library and nucleotide. These numbers were statistically assessed using the Chi-squared test of goodness of fit. For each graph in Fig 4, we compared the observed distribution to the expected based on the genomic distribution of poly (dN) tracts. All Chi-squared P values were highly significant; however, when dealing with very large numbers, P values are almost always significant. Therefore, we could not rely on P values to determine differences between cases. A better assessment of the strength of a phenomenon is its effect size [38]. To this end we calculated the Cramer’s phi effect size (ϕ_c_ = sqrt(chi2/N(k-1))). Since poly dA and dT tracts are much more abundant than poly dC and dG tracts (S2 Fig), we further normalized those numbers according to the genomic frequency of each type of tract (Fig 4).

We also randomized the data while keeping the data structure by shifting each read location by 500, 1000, and 10,000 bases. This randomization method was chosen in order to keep the distribution of reads as in the original library but to change their location in the genome.

## Supporting information

S1 Fig Base constitution surrounding both ends of the sequenced fragments. Sequence logo representation of the information content of the regions surrounding the beginning of the first read and the end of the second read for the forward (up) and reverse (bottom) strands. Note that there is absolutely no sequence information at the regions surrounding the beginning of the first reads.
(PDF)

S2 Fig Frequency of poly dN tracts in the human genome. Graph representing the number of occurrences of different sizes of poly dN in the human genome. Poly dA (blue) and poly dT (orange) tracts appear at a similar frequency in the genome and they are much more abundant than poly dC (grey) and poly dG (yellow) tracts.
(PDF)

S3 Fig Bias toward Poly dT tracts in SMART-DNA libraries. The analysis presented in Fig 4 was repeated on additional datasets and the average and standard errors of the results are shown, similarly to Fig 4. The data presented is for two HCT116 (A, D) and four HelaS3 genomic DNA libraries (B,E) sequenced by us, and for three RNA seq libraries (G) downloaded from Ramskold et al. (2012). The error bars in the random graphs (C&F) represent the standard error between three separate data randomization using a different shifting parameters (500, 1,000 and 10,000 bps).
(PDF)

S4 Fig Possible sources of bias toward poly dA/dT tracts. Endogenous sequences are shown in red whereas poly dT tracts added by the TdT enzyme are shown in blue. (A) Partially effective 3’ T tailing may result in a bias towards sequences containing genomic poly dT tracts. This type of bias can account for the large enrichment (>16%) of poly dA/dT tracts observed. (B) Using a special sequencing primer containing a poly dA tract may cause sequencing from internal poly dT tracts and may thus artificially increase the number of reads adjacent to poly dA tracts. This type of bias can account for a maximum of 4.6% of the reads since this is the estimated fraction of reads that contain genomic poly dTs. (C) Non genomic poly dT tracts may cause failure in the mapping procedure (not shown) or the introduction of a gap that artificially brings the read closer to genomic poly dA tracts. This type of bias can account for a maximum of 1.46% of the reads corresponding to the amount of reads containing a gap.
(PDF)

S1 File Supplementary Methods. (PDF)

S1 Table Basic sequencing information of all samples. (PDF)

## Abbreviations

SMART: switching mechanism at the 5′ end of the RNA transcript
NGS: next generation sequencing
ChIP: chromatin immunoprecipitation
