# Supplementary material for: Biases in the SMART-DNA library preparation method associated with genomic poly dA/dT sequences
Source: PLoS One. 2017 Feb 24;12(2):e0172769. doi: 10.1371/journal.pone.0172769 (PMC5325289; doi:10.1371/journal.pone.0172769)
Supplement: S1 Fig — Sequence logo representation of the information content of the regions surrounding the beginning of the first read and the end of the second read for the forward (up) and reverse (bottom) strands. Note that there is absolutely no sequence information at the regions surrounding the beginning of the first reads. (PDF) [file pone.0172769.s001.pdf]

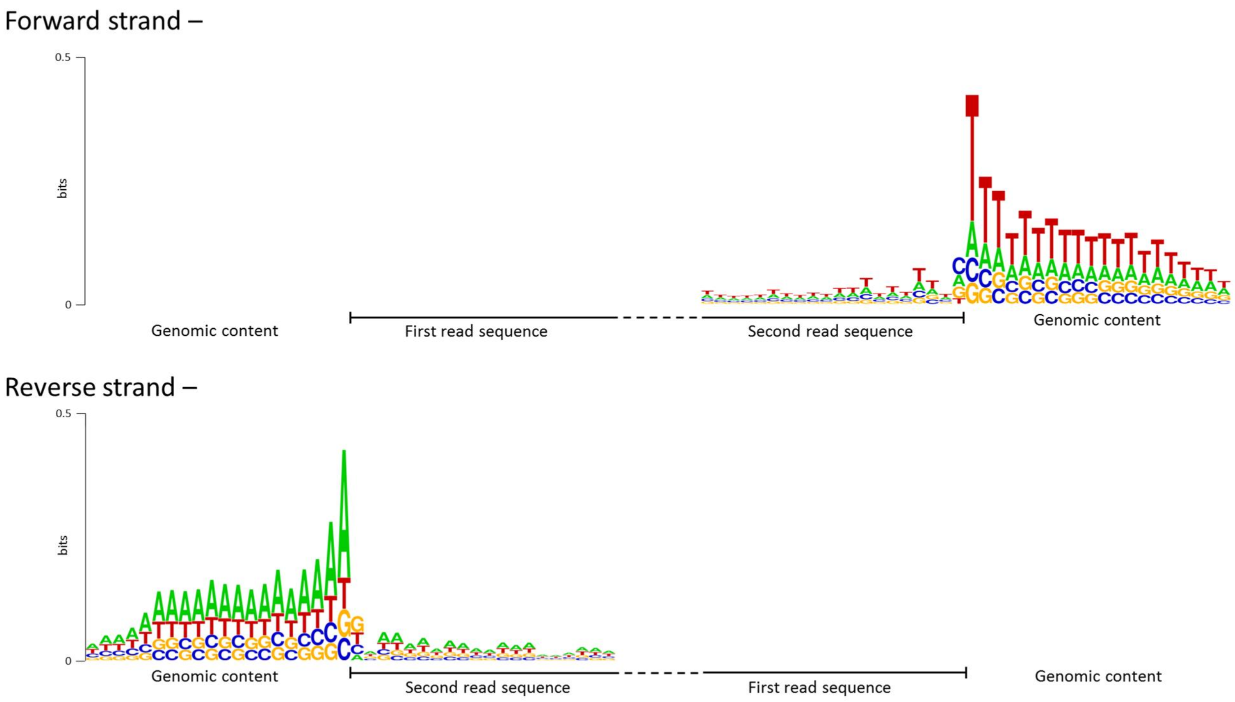

S1 Fig. ***Base constitution surrounding both ends of the sequenced fragments.*** *Sequence logo representation of the information content of the regions surrounding the beginning of the first read and the end of the second read for the forward (up) and reverse (bottom) strands. Note that there is absolutely no sequence information at the regions surrounding the beginning of the first reads.*
