# Supplementary material for: Biases in the SMART-DNA library preparation method associated with genomic poly dA/dT sequences
Source: PLoS One. 2017 Feb 24;12(2):e0172769. doi: 10.1371/journal.pone.0172769 (PMC5325289; doi:10.1371/journal.pone.0172769)
Supplement: S2 Fig — Graph representing the number of occurrences of different sizes of poly dN in the human genome. Poly dA (blue) and poly dT (orange) tracts appear at a similar frequency in the genome and they are much more abundant than poly dC (grey) and poly dG (yellow) tracts. (PDF) [file pone.0172769.s002.pdf]

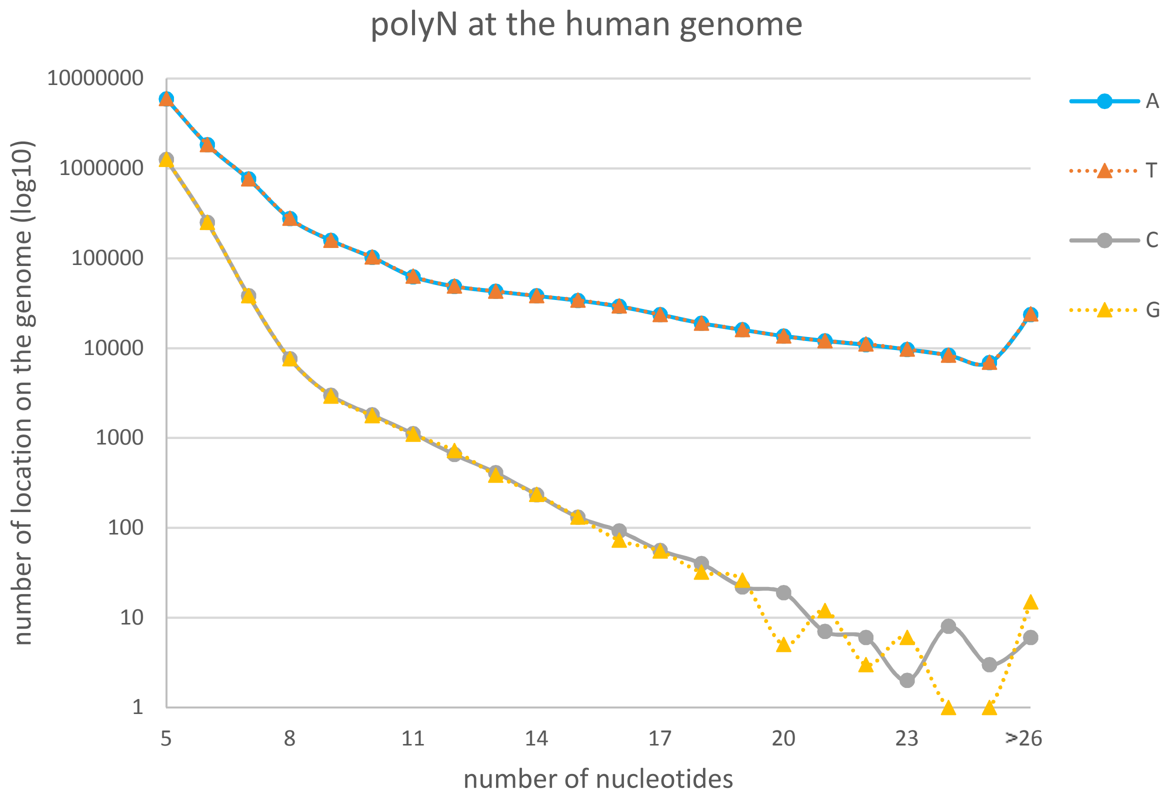

S2 Fig. Frequency of poly dN tracts in the human genome. *Graph representing the number of occurrences of different sizes of poly dN in the human genome. Poly dA (blue) and poly dT (orange) tracts appear at a similar frequency in the genome and they are much more abundant than poly dC (grey) and poly dG (yellow) tracts.*
