# Supplementary material for: Biases in the SMART-DNA library preparation method associated with genomic poly dA/dT sequences
Source: PLoS One. 2017 Feb 24;12(2):e0172769. doi: 10.1371/journal.pone.0172769 (PMC5325289; doi:10.1371/journal.pone.0172769)
Supplement: S3 Fig — The analysis presented in Fig 4 was repeated on additional datasets and the average and standard errors of the results are shown, similarly to Fig 4. The data presented is for two HCT116 (A, D) and four HelaS3 genomic DNA libraries (B,E) sequenced by us, and for three RNA seq libraries (G) downloaded from Ramskold et al. (2012). The error bars in the random graphs (C&F) represent the standard error between three separate data randomization using a different shifting parameters (500, 1,000 and 10,000 bps). (PDF) [file pone.0172769.s003.pdf]

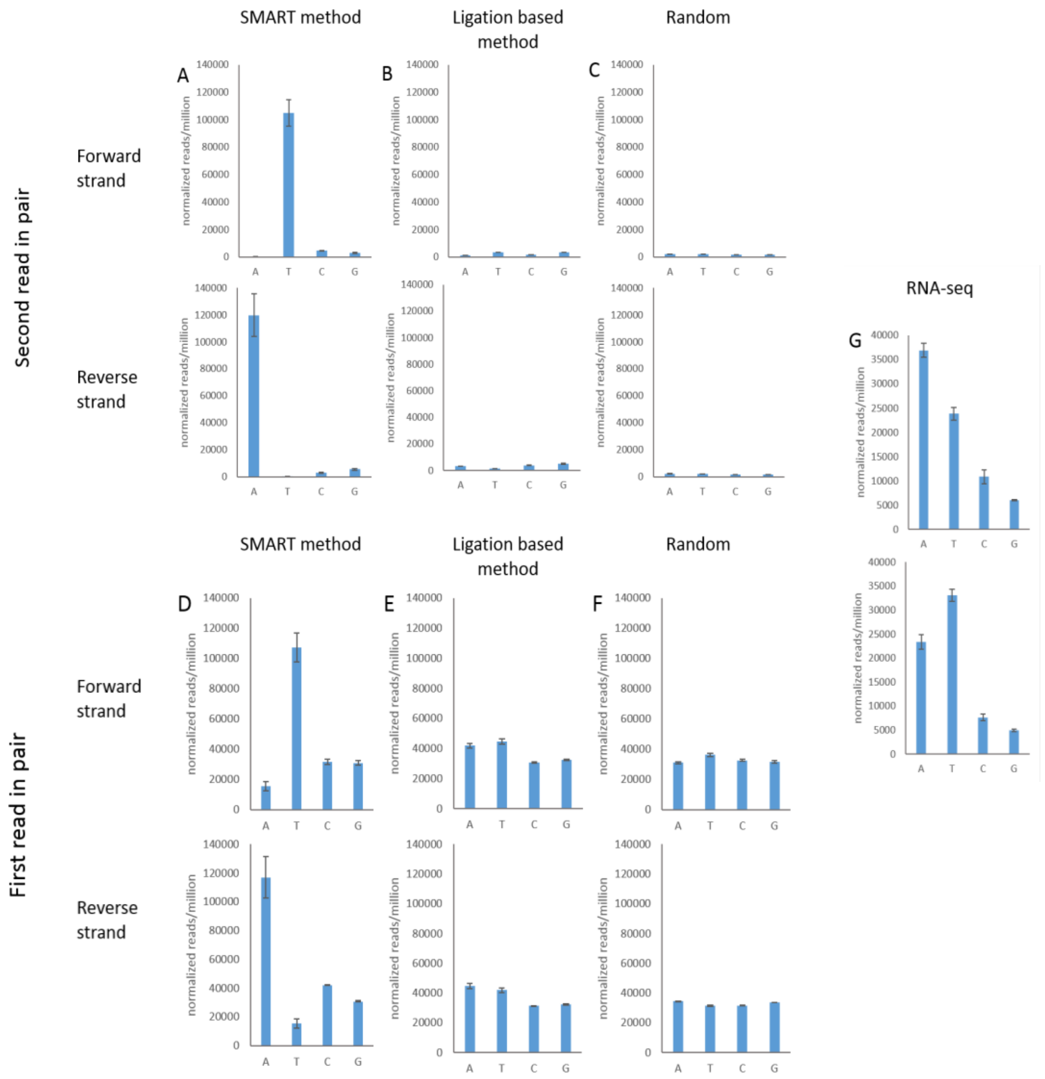

S3 Fig. Bias toward Poly dT tracts in SMART-DNA libraries. *The analysis presented in figure 4 was repeated on additional datasets and the average and standard errors of the results are shown, similarly to Figure 4. The data presented is for two HCT116 (A, D) and four HelaS3 genomic DNA libraries (B,E) sequenced by us, and for three RNA seq libraries (G) downloaded from Ramskold et al. (2012). The error bars in the random graphs (C&F) represent the standard error between three separate data randomization using a different shifting parameters (500, 1,000 and 10,000 bps).*
