# Supplementary material for: Biases in the SMART-DNA library preparation method associated with genomic poly dA/dT sequences
Source: PLoS One. 2017 Feb 24;12(2):e0172769. doi: 10.1371/journal.pone.0172769 (PMC5325289; doi:10.1371/journal.pone.0172769)
Supplement: S4 Fig — Endogenous sequences are shown in red whereas poly dT tracts added by the TdT enzyme are shown in blue. (A) Partially effective 3’ T tailing may result in a bias towards sequences containing genomic poly dT tracts. This type of bias can account for the large enrichment (>16%) of poly dA/dT tracts observed. (B) Using a special sequencing primer containing a poly dA tract may cause sequencing from internal poly dT tracts and may thus artificially increase the number of reads adjacent to poly dA tracts. This type of bias can account for a maximum of 4.6% of the reads since this is the estimated fraction of reads that contain genomic poly dTs. (C) Non genomic poly dT tracts may cause failure in the mapping procedure (not shown) or the introduction of a gap that artificially brings the read closer to genomic poly dA tracts. This type of bias can account for a maximum of 1.46% of the reads corresponding to the amount of reads containing a gap. (PDF) [file pone.0172769.s004.pdf]

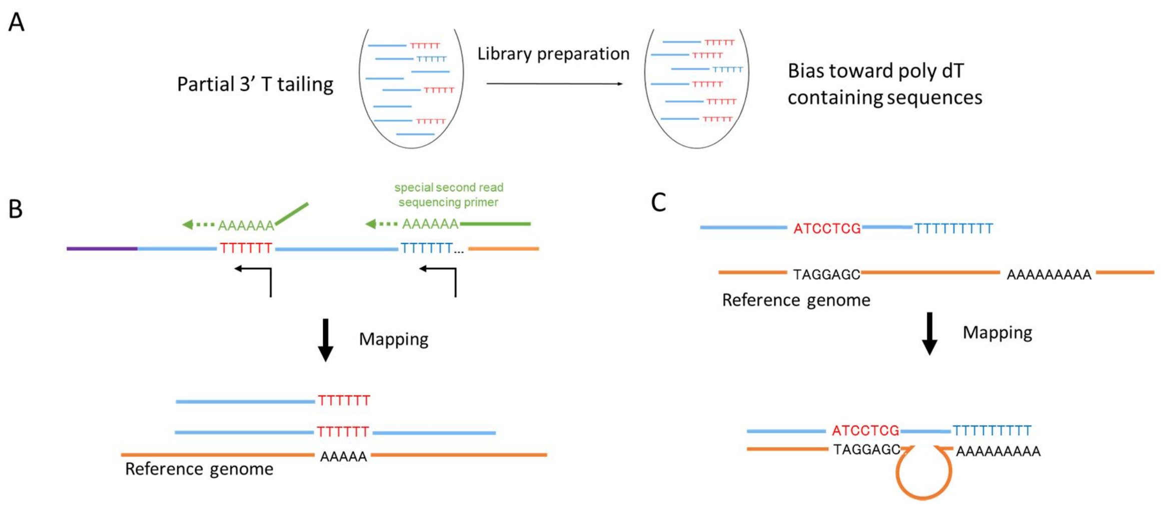

S4 Fig. Possible sources of bias toward poly dA/dT tracts. *Endogenous sequences are shown in red whereas poly dT tracts added by the TdT enzyme are shown in blue. (A) Partially effective 3' T tailing may result in a bias towards sequences containing genomic poly dT tracts. This type of bias can account for the large enrichment (>16%) of poly dA/dT tracts observed. (B) Using a special sequencing primer containing a poly dA tract may cause sequencing from internal poly dT tracts and may thus artificially increase the number of reads adjacent to poly dA tracts. This type of bias can account for a maximum of 4.6% of the reads since this is the estimated fraction of reads that contain genomic poly dTs. (C) Non genomic poly dT tracts may cause failure in the mapping procedure (not shown) or the introduction of a gap that artificially brings the read closer to genomic poly dA tracts.* This type of bias can account for a maximum of 1.46% of the reads corresponding to the amount of reads containing a gap.
