# Supplementary material for: Biases in the SMART-DNA library preparation method associated with genomic poly dA/dT sequences
Source: PLoS One. 2017 Feb 24;12(2):e0172769. doi: 10.1371/journal.pone.0172769 (PMC5325289; doi:10.1371/journal.pone.0172769)
Supplement: S1 File — (PDF) [file pone.0172769.s005.pdf]

Supplementary Methods

Mapping

Mapping to the hg19 reference genome was done with Bowtie2[1]. using default parameters with the maximum fragment length for valid paired-end alignments set at 1000. Before mapping, the first four nucleotides of each first read of the SMART based libraries was trimmed as recommended by the DNA SMART ChIP-Seq Kit (Clontech, #634865) manufacturer, using `sed '2~2s/^\(.\{4\}\)//'`. Only reads that were mapped to a single genomic location were used for further analyses. PCR duplicates were removed using the Picard tools MarkDuplicates software (http://broadinstitute.github.io/picard). Genomic locations with very large numbers of reads (>100) which likely represent repetitive sequences, were also filtered from the data.

Genomic homo-polymer identification

Poly dN genomic locations were identified using the fuzznuc program (from EMBOSS[2]). We identified all homopolymers with n≥5 bps, and determined the actual length in each genomic position.

Information content determination

The Information content of the 20 bp surrounding each end of every read was calculated using the WebLogo tool[3]. Due to limitations of this tool we randomly chose 1000 reads for drawing the webLogo. We repeated this procedure five times for each library and received essentially the same results. This procedure was also repeated for genomic regions in which at least five second reads ended at exactly the same genomic location.

Data visualization

Data visualization was done by the Integrative Genomics Viewer tool (IGV)[4] [5].

Overlap between reads and genomic homopolymers

We determined the overlap with homopolymers using BEDtools[6] and SAMtools[7] commands. We defined an overlap as a read that either overlapped or ended exactly adjacent (distance = 1bp) to poly dN tracts. This was done using the following commands - `bedtools window -r 1 -l 0 -u` and `bedtools window -r 0 -l 1 –u`, for the forward and reverse strands respectively). The number of reads overlapping or located immediately adjacent (distance = 1bp) to a poly dN tract were counted for each type of library and nucleotide, and were further normalized to reflect the number of reads per million sequenced.

These numbers were used both in figures 3 and 4.

In Figure 3, we reported the enrichment of reads overlapping or located immediately adjacent to a poly dN tract of the plus versus the minus strands. This enrichment represents the fact that in the SMART based libraries there is an over representation of reads ending near poly dA/dT tracts. Notably, the enrichment is strand specific and it is for the dT tracts in the forward strand and for dA tracts in the reverse strand. This enrichment was seen only in SMART based libraries and therefore we did not show ligation based library data in Figure 3.

In Figure 4, we present the percentage of reads adjacent to each homopolymer tract after normalizing it to the genomic occurrence of each such homopolymer ($n \geq 12$).

Statistical analysis

The significance of the over-representation of reads adjacent to the poly dA/dT tracts were assessed using the Chi-squared test of goodness of fit. For each graph in figure 4, we compared the observed distribution to the expected based on the genomic distribution of poly (dN) tracts. All Chi-squared P values were highly significant; however, when dealing with very large numbers, P values are almost always significant. Therefore, we could not rely on P values to determine differences between cases. A better assessment of the strength of a phenomenon is its effect size[8]. To this end we calculated the Cramer's phi effect size (ϕ_c=sqrt(chi2/N(k-1))).

Data randomization

We randomized the data while keeping the data structure by shifting each read location by 500, 1000, and 10,000 bases. This randomization method was chosen in order to keep the distribution of reads as in the original library but to change their location in the genome.

References

1. Langmead B, Salzberg SL. Fast gapped-read alignment with Bowtie 2. Nature methods. 2012;9(4):357-9. doi: 10.1038/nmeth.1923. PubMed PMID: 22388286; PubMed Central PMCID: PMC3322381.
2. Rice P, Longden I, Bleasby A. EMBOSS: the European Molecular Biology Open Software Suite. Trends in genetics : TIG. 2000;16(6):276-7. PubMed PMID: 10827456.
3. Crooks GE, Hon G, Chandonia JM, Brenner SE. WebLogo: a sequence logo generator. Genome research. 2004;14(6):1188-90. doi: 10.1101/gr.849004. PubMed PMID: 15173120; PubMed Central PMCID: PMC419797.
4. Robinson JT, Thorvaldsdottir H, Winckler W, Guttman M, Lander ES, Getz G, et al. Integrative genomics viewer. Nature biotechnology. 2011;29(1):24-6. doi: 10.1038/nbt.1754. PubMed PMID: 21221095; PubMed Central PMCID: PMCPMC3346182.
5. Thorvaldsdottir H, Robinson JT, Mesirov JP. Integrative Genomics Viewer (IGV): high-performance genomics data visualization and exploration. Brief Bioinform. 2013;14(2):178-92. doi: 10.1093/bib/bbs017. PubMed PMID: 22517427; PubMed Central PMCID: PMCPMC3603213.
6. Quinlan AR, Hall IM. BEDTools: a flexible suite of utilities for comparing genomic features. Bioinformatics. 2010;26(6):841-2. doi: 10.1093/bioinformatics/btq033. PubMed PMID: 20110278; PubMed Central PMCID: PMC2832824.
7. Li H, Handsaker B, Wysoker A, Fennell T, Ruan J, Homer N, et al. The Sequence Alignment/Map format and SAMtools. Bioinformatics. 2009;25(16):2078-9. doi: 10.1093/bioinformatics/btp352. PubMed PMID: 19505943; PubMed Central PMCID: PMC2723002.
8. Kelley K, Preacher KJ. On effect size. Psychological methods. 2012;17(2):137-52. doi: 10.1037/a0028086. PubMed PMID: 22545595.
