# Supplementary material for: Biases in the SMART-DNA library preparation method associated with genomic poly dA/dT sequences
Source: PLoS One. 2017 Feb 24;12(2):e0172769. doi: 10.1371/journal.pone.0172769 (PMC5325289; doi:10.1371/journal.pone.0172769)
Supplement: S1 Table — (PDF) [file pone.0172769.s006.pdf]

S1 Table. Basic sequencing information of all samples.

#	Cell type	Library preparation method	Read length	Number of reads	Used in figure
1	HCT116	SMART	2x50	75853461	Fig. 2, 3, 4;A,D
2	HCT116	ligation	2x50	40859208	Fig. 4;B,E
3	HCT116	SMART	2x50	207727749	Fig. S2;A,D
4	HCT116	SMART	2x50	44753332	Fig. S2;A,D
5	HeLa-S3	ligation	2x38	42750844	Fig. S2;B,E
6	HeLa-S3	ligation	2x38	40970214	Fig. S2;B,E
7	HeLa-S3	ligation	2x38	35214656	Fig. S2;B,E
8	HeLa-S3	ligation	2x38	35877224	Fig. S2;B,E
